# Predicting State Anxiety Level Change Using EEG Parameters: A Pilot Study in Two Museum Settings

**DOI:** 10.3390/brainsci15080855

**Published:** 2025-08-11

**Authors:** Maria Elide Vanutelli, Annalisa Banzi, Maria Cicirello, Raffaella Folgieri, Claudio Lucchiari

**Affiliations:** 1 Department of Psychology, University of Milan-Bicocca, 20126 Milan, Italy; maria.vanutelli@unimib.it (M.E.V.);; 2 Department of Philosophy “Piero Martinetti”, Università degli Studi di Milano, 20122 Milan, Italy; annalisa.banzi@unimi.it (A.B.); raffaella.folgieri@unimi.it (R.F.)

**Keywords:** BCI, museum, well-being, stress, anxiety, theta-beta ratio

## Abstract

**Background:** Museums are increasingly being recognized not only as cultural institutions but also as potential resources for enhancing psychological well-being. Prior research has shown that museum visits can reduce stress and anxiety, yet there is a pressing need for evidence-based interventions supported by neurophysiological data. While neuroscientific studies suggest a combined role of emotional and cognitive mechanisms in aesthetic experiences, less is known about the neural predictors of individual responsiveness to such interventions. **Methods:** This study was conducted in two Milan-based museums and included an initial profiling phase (sociodemographic information, trait anxiety, perceived stress, museum experience), followed by pre- and post-visit assessments of state anxiety and mood. Electrocortical activity was recorded via a portable brain–computer interface (BCI), focusing on the theta/beta ratio (TBR) as a marker of cortical–subcortical integration. **Results:** Museum visits were associated with significant improvements in mood (M = 1.17; *p* < 0.001) and reductions in state anxiety (M = −6.36; *p* < 0.001) in both arts and science museums. The baseline TBR predicted the magnitude of state anxiety change, alongside individual differences in trait anxiety and perceived stress. **Conclusions:** These findings support the idea that aesthetic experiences in museums engage both emotional and cognitive systems, and that resting state neurophysiological markers can help forecast individual responsiveness to well-being interventions. Such insights not only contribute to existing knowledge about the cognitive and emotional processes during aesthetic fruition, but could also guide future applications of personalized interventions in museum settings, further integrating cultural participation with mental health promotion.

## 1. Introduction

### 1.1. The Healing Gaze: Art Fruition, Stress Relief, and the Brain

Contemplation and engagement with visual art have been increasingly recognized for their beneficial effects on psychological well-being. The typical psychological responses to an aesthetic experience, defined as the emotional response to the intrinsic, non-utilitarian qualities of a piece of artwork, are increased mindfulness and positive mood [1,2]. In fact, research indicates that both the creation and appreciation of art can serve as preventive interventions, reducing psychological distress, enhancing self-awareness, promoting behavioral change, and modulating physiological responses such as heart rate, blood pressure, and cortisol levels [3,4,5,6]. All these indices describe a psychophysiological state characterized by reduced stress and a higher sense of relaxation. However, art fruition can also elicit feelings of privilege, inspiration, and emotional insight, even in vulnerable individuals such as the elderly or those with chronic mental illness [7,8]. For example, in a study of 300 hospital patients and care home residents, object handling sessions have been shown to foster social interaction, increase vitality, stimulate tactile engagement, and promote a sense of identity and self-worth [9,10].

As detailed in the review by Mastandrea and colleagues [2], and taking inspiration from the results obtained in the music fruition field, the experience of pleasure in visual art reception could emerge from a dynamic interplay of processes, including (1) the affective resonance with the emotional valence expressed by the artwork; (2) the cognitive appraisal of negative emotional content in a safe and sublimating space; (3) the consequent regulation of emotional responses; (4) the engagement in aesthetic appreciation and active judgment. To explain these effects, both emotional and cognitive mechanisms have been considered including memory, identity reinforcement, and meaning-making [11,12].

Neuroscientific investigations have shed light on the neural underpinnings of aesthetic experiences. Visual art appreciation is associated with brain activations in areas devoted to higher level emotional processing, decision-making, and the reward system, such as the orbitofrontal cortex (OFC), the ventromedial prefrontal cortex (vmPFC), the anterior cingulate cortex (aCC), and the striatum [2,13,14,15], reinforcing the idea that both cognitive and affective processes are engaged during aesthetic appreciation [16].

### 1.2. Healing Spaces: Psychological and Neural Effects of Museum Collections

In recent years, museums have been designated as the new spaces of choice for promoting visitor well-being, particularly in the context of mental health [9,17,18,19]. Museum-based programs offering activities such as guided tours, object handling, mindfulness, and creative workshops have been developed for a range of populations, including older adults, individuals with dementia, and psychiatric patients [8,20,21], but also the general public [4]. Qualitative and quantitative studies consistently report that participation in museum activities can improve subjective well-being, reduce anxiety, and enhance mood [4,8,9,22]. Increased optimism, hope, and enjoyment have been reported as contributing factors, along with the reduction in social isolation and the opportunity to learn new concepts and skills, with subsequent enhanced self-esteem and sense of identity [7].

A notable example is the Museums on Prescription initiative, where museum-based social prescribing was associated with significant pre and post improvements in emotional states such as absorption, enlightenment, and cheerfulness [8]. Importantly, the participants also valued opportunities to engage with curators and artifacts, suggesting that co-creative, interactive approaches maximize therapeutic potential. These findings support the reconceptualization of museums as inclusive, community-based environments capable of facilitating health-related outcomes beyond clinical settings [20,23].

Although still a growing field, early evidence from neuroaesthetics and museum studies suggests that the museum setting itself may amplify the neural effects of aesthetic experience. The architectural and curatorial features of museums, characterized by spatial focus, reduced cognitive noise, and multisensory richness, are hypothesized to support attentional engagement, emotional regulation, and reflective thought [2,4,24,25,26,27].

Neuroimaging research conducted in ecologically valid environments indicates that museum visits can modulate the neural activity associated with stress and emotional processing. For instance, viewing figurative art in situ can lead to decreased physiological arousal [5] and improved mood, particularly when context enhances the understanding of the artwork [28].

While imaging methods can provide meaningful insights into aesthetic preference in experimental contexts, electrophysiological data seem more promising in ecologically valid settings, such as a museum visit, when conducted with portable devices.

### 1.3. Electrophysiological Markers of Anxiety

Anxiety and stress are known to correlate with attentional control and executive functions. In the context of cognitive performance anxiety (CPA), top-down attentional control, mediated by the prefrontal cortex (PFC), fails to override the stress-induced processing of threat-related information. This disruption occurs because attentional control is governed by a dual-process system: bottom-up (posterior/subcortical) and top-down (anterior/prefrontal) networks. Anxiety disturbs the balance between these systems, enhancing stimulus-driven attention and consequently increasing the detection and processing of threat-related stimuli [29].

Several studies investigating EEG slow/fast rhythm dynamics suggest that the spontaneous change in the ratio between theta (4–7 Hz) and beta (13–30 Hz) might indicate significant shifts in emotional/cognitive spheres. In particular, the theta/beta ratio (TBR) seems to reflect cortical–subcortical interactions, specifically those involved in emotional states, dispositional affective traits, and emotion regulation [30]. An elevated TBR has been observed in the inattentive subtype of attention deficit disorder (ADD) and Attention Deficit/Hyperactivity Disorder (ADHD), reinforcing its association with stable affective traits (e.g., anxiety and behavioral inhibition), incentive motivation, risky decision-making, and impaired inhibitory control (i.e., attentional bias to threat) [31]. Similarly, a low frontal TBR has been shown to predict resilience under stress-induced impairments in attentional control. For example, performance on an emotional go/no-go task revealed an inverse correlation between frontal TBR and the fearful modulation of response inhibition, as well as with self-assessed indices of attentional control and anxiety. Higher slow/fast EEG ratios have also been linked to reduced adaptive anxiogenic responses, characterized by more inhibited reactions to fearful, rather than happy, facial expressions [32]. TBR is therefore considered an electrophysiological marker of executive cognitive control, which moderates the effects of CPA-like effects on attentional control. Higher TBR scores then indicate greater slow-wave power (i.e., subcortical control) relative to fast-wave power (i.e., cortical control).

In line with prior research utilizing portable EEG systems, we corroborate the finding that anxiety states elevate theta oscillations in the PFC and that the eyes-closed condition, rather than the eyes-open condition, more reliably predicts anxiety levels following the task [33]. Thus, the TBR has been established as a reliable marker of attentional control over emotional stimuli, positioning it as a promising tool for investigating cognitive–affective regulation, particularly in contexts involving stress and anxiety.

Previous research already tested the feasibility and usability of EEG data in museum contexts [34] and highlighted promising results related to visitors’ engagement [35]. However, the identification of neural signatures related to the effectiveness of interventions aimed at stress reduction needs further investigation.

### 1.4. The Present Study

Despite the growing body of evidence supporting the beneficial impact of museum experiences on psychological and neurocognitive well-being, the literature still lacks approaches for tailoring such interventions to individual needs by identifying those most likely to benefit. This limitation reflects a broader gap in our understanding of the neural markers that predict responsiveness to art-based experiences. The aim of this study was to address this gap. It was not only meant to explore the beneficial effects of museum visits on stress reduction, but also to identify the neural correlation underlying individual differences in the impact of museum visits. This could potentially support the findings from clinical populations, but also hopefully extend our knowledge on the general public, supporting the broader application of art-based interventions on psychological well-being [18,36].

The present study is part of a larger protocol called the ASBA Project (Anxiety, Stress, Brain-friendly Museum Approach) [17], which integrates electrophysiological and psychometric assessments to evaluate the effectiveness of targeted museum experiences in reducing perceived stress. Launched in October 2022, the project was proposed in both fine art and natural history museum settings in Milan and Turin and was targeted at both general visitors and museum staff [4].

The participants were involved in an interactive museum visit. Before and after the intervention, both self-reported measures (perceived stress) and electrophysiological responses were recorded using a brain–computer interface (BCI) device. By combining subjective and psychophysiological measures, we aim to provide further insight into how cultural participation within museum contexts contributes to resilience, mood regulation, and overall mental health.

## 2. Materials and Methods

### 2.1. Participants

Thirty-five healthy adults (M_age_ = 32.29, SD = 7.8) aged 18–54 participated in the experiment (see Table 1) either at the Modern Art Gallery (MAG) or the Natural History Museum (NHM; Milan, Italy). Eighteen of them visited the MAG and seventeen visited the NHM. The sample was composed of 26 women (74%) and 9 men (26%). More than 54% of them reported visiting museums more than 6 times a year; 21.5% and 19% have an attendance of 3–5 and 1–2 times a week, respectively. Just 5.1% never visit a museum throughout the year.

The recruitment process entailed posting the call for applications on the websites of the participating universities, as well as on local government and museum websites. To include people of various ages and social, economic, and cultural backgrounds, the project received the most publicity possible. Through the websites, participants could access the project page, schedule the visit date, and read the informed consent information. The consent information explained all the procedures in detail, along with the objectives and the risks of the study. It also included a detailed section explaining the participant’s right to withdraw from the study at any stage, along with the privacy policy. Then, upon agreeing to the terms, the participants completed the initial demographic questionnaire. This questionnaire was used to gather personal data and determine inclusion and exclusion criteria. The participants then filled out the psychological traits questionnaires and were assigned to a given study session (see the Procedure Section).

Inclusion criteria:

- Being 18 years old or older.
- Accepting the conditions for participation and having signed the informed written consent.

Exclusion criteria:

- Insufficient language skills in understanding verbal assignments and interacting with presenters and peers.
- Current diagnosis of a disease of psychiatric or neurological nature.
- Uncorrected visual or hearing impairment.
- Another medical condition that could negatively affect the activities to be performed.

This study was conducted with the understanding and written consent of the participants, who had been informed of the research procedures and purposes according to the Declaration of Helsinki and with the approval from the local Ethical Committee (University of Milano-Bicocca; protocol code: 733).

### 2.2. Procedure

#### 2.2.1. Procedure Phase 1: Welcoming Procedures

On the day of the session, the participants were welcomed in a designated area of the museum and check-in was carried out to verify the correct completion of the questionnaire and to sign the informed consent form. As soon as all of the participants in the session arrived, they were led to a boardroom in the museum used for the presentation of the study. Here, the most important information about the study was reiterated and the professionals and researchers involved were introduced, together with a brief presentation of the brain–computer interface technique. The pre-assessment, museum experience, and post-assessment phases were all concluded within the same day.

#### 2.2.2. Procedure Phase 2: Pre-Visit Assessment

Then, pre-treatment questionnaires were administered, which were designed to measure state anxiety levels (SAI) and mood (VAS) (see below). EEG measurements were recorded for two minutes under quiet conditions. The participants could choose any seat in the large hall to encourage comfort and self-confidence.

#### 2.2.3. Procedure Phase 3: The Visit

Small groups (7 to 10 participants) were then formed and accompanied by a museum expert in an interactive visit to some museum’ rooms. Space and time allocated to each group were standardized so that all had a similar experience. Each group had 90 min to conclude the visit.

#### 2.2.4. Procedure Phase 4: Debate

Following the visit, a discussion on the experience was led by the conductor after moving the chairs into a circle to facilitate sharing. In this space, each participant could share their feelings and opinions on what they had experienced, including which artifact (or part of it) they had focused on. In the case of the art museum, the aim was to place the work in its historical and aesthetic context and to link this information to what participants reported about their experience. In the case of the science museum, the characteristics of the various animals and plant species that were present in the dioramas were illustrated, together with geographical coordinates to allow participants to better understand their experience and to acquire knowledge. This phase lasted 30 min.

#### 2.2.5. Procedure Phase 5: Post-Treatment Assessment

At the end of the discussion, the participants were accompanied back to the boardroom, where the EEG measurements (through BCI) were recorded for two minutes under quiet conditions. The participants were then asked to fill in the post-treatment questionnaires, which were identical to those of the pre-experience phase, except for an additional space at the end of the document for them to make a general comment on the experience and to give it a title as if it were a movie.

#### 2.2.6. Procedure Phase 6: Debriefing

**Figure 1:**
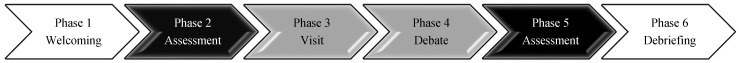
Graphic representation of the experimental procedure.

At the end of the post-experience evaluation, the participants could ask further questions to the professionals and researchers, as well as receive further information on future developments of the study or where to find information for further investigation (see Figure 1).

### 2.3. Instruments

#### 2.3.1. Recruitment Questionnaire

For each new slot available, one month before the beginning of the sessions, the calendar for registration was made available to the public. The day before the session, the registration closed to allow the researchers to check for eligibility and completeness of the data. The response rate was highest especially in the first available slots, decreasing throughout the following slots. However, each session never hosted less than 10 participants.

To determine the participants’ demographic information and relationship with the museum, the questionnaire included sociodemographic information such as gender (female, male, other, prefer not to say) and age, along with information related to participants’ habits with respect to museum visits (annual attendance at any type of museum with four choice ranges: never; 1–2 times; 3–5 times; >6 times) and their preferences (interest in art and science museums on a Likert scale from 1—not at all to 10—very much).

The State and Trait Anxiety Inventory (STAI; [37]) is the most widely used tool in the scientific literature for the psychometric measurement of anxiety. The theory of state and trait anxiety, which distinguishes between current anxiety and readiness for anxious reaction as a personality trait, now appears to be supported by both clinical evidence and numerous experimental studies. The brevity of the questionnaire and the simple formulation of the items makes it very easy to administer and ensures good validity of the scores obtained. The STAI questionnaire consists of two separate scales to measure two distinct anxiety constructs: State Anxiety Inventory (SAI) and Trait Anxiety Inventory (TAI). Both scales consist of 20 statements where participants are asked to describe how they generally feel, on a Likert scale ranging from 1 (not at all) to 4 (very much). In the recruitment questionnaire, just the TAI was administered. The SAI, instead, was applied during the pre-/post-assessment phases (see ahead).

The Perceived Stress Scale (PSS; [38]) is the most widely used psychological instrument for measuring the perception of stress. It is a measure of the degree to which situations in a person’s life are rated as stressful. The items were constructed to intercept the degree to which people find their lives unpredictable, uncontrollable, or overloaded. The scale also contains a series of direct questions about current levels of perceived stress. Both the items and the alternatives are easy to understand. In addition, the questions are general and thus free of content specific to any subpopulation. The PSS questions concern feelings and thoughts related to the last month. For each item, people are asked to indicate how often they felt a certain way, from 0 (never) to 4 (very often). Scores higher than 27 are considered high, while people with a score lower than 14 are considered to have no stress.

#### 2.3.2. Pre- and Post-Experience Assessment Questionnaires

SAI: To assess the changes in State Anxiety, the SAI scale was administered both before and after the museum visit (pre- and post-experience assessment) following a pre/post protocol similar to Binnie [22], who reported positive results after a museum visit. The scale can detect short-term changes in anxiety levels thanks to its adequate psychometrics parameters, as shown in many previous studies (e.g., [39,40,41]). As mentioned above, the scale includes 20 statements where participants are asked to describe how they feel in the present moment on a Likert scale ranging from 1 (not at all) to 4 (very much).

MOOD-VAS: Six visual–analog scales (VAS) [42] were presented to assess participants’ moods and states of mind before and after the museum visits. The first question was about general mood, i.e., “How do you rate your mood right now?” with a choice among 10 steps from 1 (negative) to 10 (positive). The next 5 questions required assessing the intensity of certain states of mind experienced at the present moment, from 1 (absent state) to 10 (very present state), and specifically stress, mental clarity, contentment, calmness, and restlessness.

Brain–computer interface (BCI): A BCI was used to measure real-time brain activity and potentially translate it into commands that can be used to control devices [43]. In medicine, BCIs have demonstrated their potential in helping people with various conditions. For example, BCIs have been used to help people with paralysis regain some level of control over their movements by translating their brain activity into control signals for prosthetic limbs [44,45]. BCIs have also been used to help people with epilepsy detect and prevent seizures [46,47]. Like traditional EEG, BCI systems are powerful and comfortable tools to record biosignals from the scalp. However, BCI are generally wireless and more wearable than traditional EEG. Furthermore, we used a device based on dry electrodes to reduce related negative effects on participants’ experience in a real-world setting like a museum. Considering the research aims, it was important that participants could have a meaningful and enriching experience. Thus, we used a commercial BCI device, MUSE (InteraXon Inc., Toronto, ON, Canada) headband, that is easily wearable and provides 4 electrodes (2 frontal and 2 temporal-posterior). Furthermore, only resting pre- and post-experience EEG activity was recorded, so it did not interfere with the visit experience. Each device was then connected to a smartphone via Bluetooth and data were collected using the Mind Monitor application developed by James Clutterbuck. Participants’ brain activity was then recorded in a comfortable seat inside a room of the museum without interacting with other participants. Data were sampled at 256 Hz, applying a 50 Hz notch frequency filter. Mind Monitor processes raw data by applying a Fast Fourier Transform to obtain the distribution of the brain activity in different frequency bands. The logarithm of the Power Spectral Density (PSD) of the raw EEG of each channel is applied. Though automatic EEG processing was computed using Mind Monitor, EEG raw data were visually inspected to avoid including bad traces in the analysis.

The EEG sessions lasted 2 min each (pre and post experience): 1 min eyes open and 1 min eyes closed. Participants were asked to stay relaxed, maintaining a comfortable position and limiting movement and distractions. The cue to close the eyes was given one minute after the beginning of the recording session using a soft jingle. To reduce the effect of eye blinks, we used only the eye-closed EEG recordings in our analysis.

We took every precaution to ensure the best possible signal, for example, by cleaning the skin before putting on the MUSE, adjusting the device as closely as possible to the participant’s head, and allowing them time to familiarize themselves with the situation and instructions. Before recording, the optimal impedance level was achieved and constantly monitored by the researcher. The Mind Monitor application uses specific codes to mark Bluetooth connection as well as other technical issues, so that bad data points may be excluded from analysis. This permitted us not to exclude the whole trace in the presence of a few bad data points.

## 3. Results

The analyses have been conducted using SPSS Statistics version 28.0.1.1 (IBM SPSS Statistics for Windows. Armonk, NY, USA: IBM Corp).

### 3.1. Descriptive Statistics

First, descriptive statistics have been performed to give a picture of the participants’ profiles. Most of them reported visiting museums regularly and to be interested in the subjects of art or science. Regarding psychological traits, Table 1 reports participants’ trait anxiety levels, the Perceived Stress Scale (PSS) scores, and a self-evaluation of their own stress.

All participants reported a moderate self-reported level of stress and anxiety. No significant differences were found between the two samples for any of the trait variables measured, as assessed by *t*-tests for independent samples (all *p* > 0.05).

### 3.2. Changes in Anxiety Levels After the Visit

To assess whether visiting museums had caused changes in state anxiety or mood levels, paired-sample *t*-tests were conducted for measures of interest including SAI and VAS. Since the purpose of the study was not to compare the two museums with each other, we did not perform a mixed model.

The *t*-tests revealed significant results for all the dependent variables: SAI, mood, stress, mental clarity, contentment, calmness, and restlessness (see Table 2).

In detail, anxiety levels, stress, and restlessness proved to be significantly decreased after the activities in the museum, while mood, mental clarity, contentment, and calmness were increased (see Table 3).

**Figure 2:**
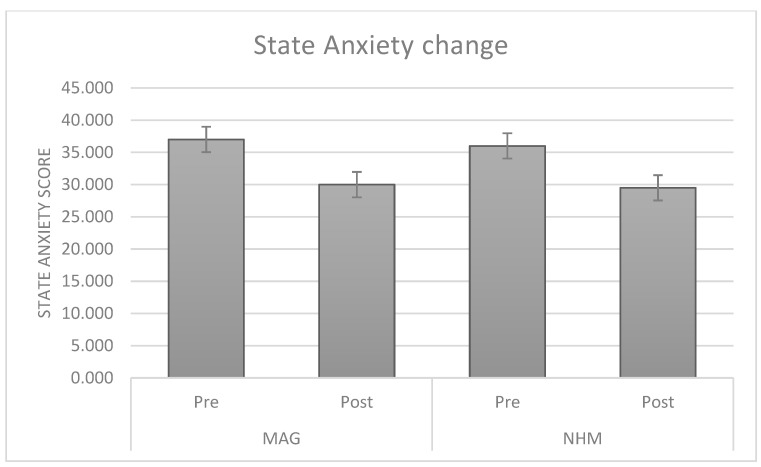
Histograms of the SAI scores as recorded before and after the visit to the arts (left) and science (right) museums.

The visit was able to significantly reduce state anxiety in both museum settings (see Figure 2).

### 3.3. BCI and Psychological Measures Pre and Post Changes

A correlation analysis was performed to explore if different psychological variables and the EEG measurements showed significant relationships. Both state and trait measurements were input to analyze possible mediating effects. For the EEG measurements, the power of each EEG range was considered (delta, theta, alpha, beta, and gamma) in pre and post assessment, as well as the theta/beta ratio (TBR) that was chosen based on the literature as the main state anxiety index (see Section 1.3). The Pearson r statistics revealed that the pre-visit SAI correlated only with PSS scale, while the post-visit SAI correlated only with the trait anxiety score. Then, delta scores were also computed to obtain SAI and TBR change after the intervention (ΔSAI = SAI_post_−AI_pre_; ΔTBR = TBR_post_−TBR_pre_). The post and pre anxiety difference (ΔSAI), and thus the change in SAI after the visit, was correlated only with the post and pre TBR difference (ΔTBR). No other EEG value correlated with state anxiety.

Starting from the above data, a linear regression analysis was run using SAI change (ΔSAI) as the dependent variable and trait anxiety (TAI), trait perceived stress (PSS), and TBR change (ΔTBR) as the predictors.

**Figure 3:**
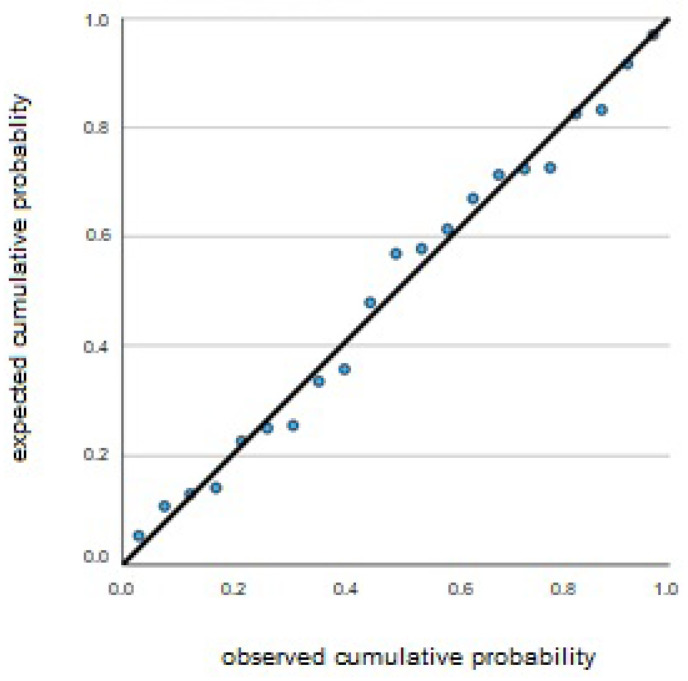
Normal PP regression graph (dependent variable DeltaSAI).

As Table 4 shows, the best predictor of the state anxiety changes (ΔSAI) is the change in TBR (ΔTBR), that is the difference between the average power of the theta/beta ratio pre and post experience (R^2^ = 0.441; see Figure 3).

However, trait anxiety and the level of perceived stress are, as predicted, also associated with the change in state anxiety. In particular, the higher the state anxiety, the greater the change that can be expected in terms of state anxiety. On the other hand, the baseline level of perceived stress (PSS score, which refers to the previous month) seems to have an inverse relationship: the lower the stress, the higher the change.

## 4. Discussion

The aim of the present study was twofold: the first goal (I) was to explore the beneficial effects of museum visits on stress reduction; the second goal (II) was to identify the neural correlation underlying individual differences in the impact of museum visits.

Starting with the first aim, the analyses conducted on state anxiety before and after the visit demonstrated that all the dependent variables improved, with increased calmness and better mood, and a decrease in perceived stress, anxiety, and restlessness. Thus, we can conclude that this study supports previous evidence conducted in museum settings that report a greater well-being effect in relation to various experiences [4,8,9,22].

A particularly compelling outcome has been found in relation to the second aim and lies in the predictive value of the theta/beta ratio (TBR) on visitors’ well-being. Despite the exploratory nature of this analysis, the results indicate that resting state EEG measurements can forecast the magnitude of anxiety change after the intervention. TBR was selected for its capability to indicate significant shifts in emotional/cognitive spheres through cortical–subcortical interactions [30]. In fact, a modulation of TBR has been found in several mental processes where these two aspects strongly interact, including motivation, decision-making, and inhibitory control [31]. This finding, therefore, puts the results in line with the previous literature that had already discussed the importance of cognition and emotion in processes related to aesthetic experience [11,12,16]. However, it complements it by suggesting a new marker that can easily be used in future studies for tracking well-being. Besides TBR, perceived stress and trait anxiety proved to predict the outcome of the intervention. This last result is very valuable as it suggests that there is much room for developing future interventions that can take into account individual differences and potential benefits, including discriminating between different intervention types.

An apparently incongruent result is the disassociation between trait anxiety and perceived stress in predicting state anxiety change. In fact, greater changes in state anxiety were predicted by lower perceived stress and higher trait anxiety. Although the two constructions are surely related, there are some differences that could explain these different directions. Trait anxiety, while representing a stable predisposition, may enhance the individual’s sensitivity to situational changes. For example, Schwerdtfeger et al. [48] reported that a brief “best possible selves” intervention before a stress task elicited adaptive physiological responses in individuals with a high trait anxiety. Thus, individuals with a higher trait anxiety may experience greater relief when exposed to restorative, positive experiences. In contrast, perceived stress often reflects lifestyle and a more chronic, generalized overload and lack of control, which may counteract responsiveness to short-term interventions. In a study by Pizzagalli and colleagues [49], stress scores, as measured precisely with the PSS, predicted reduced hedonic capacity even after controlling for general distress and anxiety symptoms. These results are in line with subclinical data showing an association between stress and anhedonia, as well as stress and depression. Future studies should better explore the differential effects of museum interventions on anxiety and depression symptoms.

## 5. Conclusions

This study is part of a larger research activity, the ASBA project [17], which aims to validate museums as privileged places for self-care and to collect and share data and methodologies that can be used in a variety of contexts. So, on the one hand, it aims to put the museum at the center of community life, thus creating a socio-cognitive space to live enriching experiences. On the other hand, it aims to harness the inherent power of art and culture on the human mind as well as on relative health.

The results of this study show how museum activities can actually be effective in promoting well-being. Not only was the intervention effective in reducing state anxiety, but it was also compatible with normal museum activities and highly appreciated by participants and instructors. This reinforces the idea that neuroscience and brain technology are not destined to remain within research laboratories but will increasingly become part of everyday life [50,51], performing a variety of functions. Future research may also include artificial intelligence-based learning models that maximize the BCI device’s ability to detect even small variations in a single person or group. It is therefore necessary to carry out more and more real-world studies to validate these neuroscientific applications, both in terms of effectiveness (e.g., measurement of state anxiety) and in terms of usability and robustness (e.g., Bluetooth signal stability and signal-to-noise ratio).

Furthermore, it was possible to identify a neural marker (TBR) that could potentially signal the amount of benefit that one can obtain, paving the way to a huge variety of future tailored interventions. While the TBR specificity for emotional control is disputed (see Clarke et al., 2019 [52]), TBR can effectively capture the cognitive and emotional characteristics we focused on in our study [30,31]. More complex indices, also analyzing interhemispheric asymmetries, particularly in the alpha band, and using specific tasks could be used instead. However, as we wanted to use a simple and quick EEG recording, we believe that TBR is an excellent option, since it reliably mirrors the cognitive balance between the cognitive and emotional control systems, which we expected to elicit with our museum-based activities (Kaushik, 2024) [53]. Similarly, it must be considered that the change in state anxiety measures precisely the transition from a state of alertness, which in turn is a function of trait parameters such as stress and anxiety as well as the context, to a state of emotional and cognitive comfort. That is why we expected to find significant correlations between TBR and state anxiety variations.

Finally, the present study has shown not only how art museums can be considered ideal places to promote individual well-being, as other studies have shown [2,54], but also that science museums can pursue the same purpose, as suggested by a limited number of previous studies (e.g., French et al. [55]).

This study is not without limitations. First, it was not possible to collect EEG measurements from all participants. Due to organizational and resource issues, but also personal availability (many participants were elderly and not particularly ready for brain technology; many others were distrustful), only a small sub-sample of 35 participants successfully participated in EEG recording. Furthermore, the participants were not randomized between the two museums. Another inherent limitation of this study is that it does not have a control group, so it is not possible to exclude that the effects revealed, in particular the benefits on subjective well-being, are not due to factors other than museum experience. Altogether, the above considerations reduce the statistical power of our analysis, hence the exploratory nature of the present study. 

Regarding our results, two aspects deserve further attention in future studies. First, our regression model has a moderate explanatory power of the regression model (R^2^ = 0.441), suggesting that other factors can predict state anxiety change that our study could not detect. Second, the inverse relationship between the score of the Perceived Stress Scale (a trait value) and the state anxiety change. Since we observed that trait anxiety has a direct correlation with state anxiety change, one could expect to find the same direction also for perceived stress. Instead, it seems that the two traits’ values are dissociated. This datum needs further investigation, because traits values can be useful to direct people toward personalized treatment, and thus is important to know if museum experience is more effective for people with high or low perceived stress.

Future studies expanding the sample size will allow for the use of more in-depth and accurate statistics, for example, by comparing the specific effect of the museum setting (artistic vs. scientific) and also appropriately correcting for the effect of multiple comparisons. The data presented here must certainly be considered in the context of these limitations, which circumscribe both the statistical power and the generalizability of the results. It would also be interesting to compare the effects obtained in museums with those obtained in other settings, particularly ones not generally associated with well-being or comfort, such as a school context. Finally, as suggested above, future studies should test the predictive power of other EEG metrics. In particular, we used only baseline values (pre and post experience), but it could be possible to devise brief tasks, for instance tasks that are able to increase alertness and/or solicit executive control, during which collecting EEG data, in a way more similar to laboratory tasks but in a real-world setting. It would be possible then to collect significant data about individual EEG responsiveness and test more complex statistical models to predict state anxiety change.

However, given the promising results, the study will be continued with the hope of obtaining a much larger sample in a relatively short time. Moreover, the group-based nature of the activities and the reliance on self-report measures may constrain the generalizability of our findings. Nevertheless, we provided initial evidence that the protocol can be successfully adapted and implemented across different museum contexts. This flexibility suggests that museums in the future could integrate such interventions into their regular programming, offering accessible tools to enhance visitors’ well-being and foster deeper engagement.

More generally, based on our results, it will be possible to design more accurate studies, also with a view to using the TBR index in clinical settings. In particular, future studies could focus on understanding the relationship between TBR (as well as other EEG indices) and various symptoms, including anxiety and depression. In particular, if our data are confirmed and extended to other settings and clinical populations, it will be possible to use these indices more systematically to evaluate the effectiveness of treatments and adjust therapies accordingly. The idea behind this study is to provide tools and methods that can be used in real-world settings and are at the same time inexpensive, easily implementable, and reliable. This would allow for increasingly personalized therapies in the psychiatric field and generalize the use of technology (including artificial intelligence) in a variety of professional contexts, even in the absence of the technical and economic resources generally required for the use of laboratory equipment.

To conclude, we believe that this study is of particular importance in bringing applied neuroscience closer to contexts such as museums, where it could play a key role in improving well-being. Also, its importance also lies in contributing to the knowledge about the emotional and cognitive processes underlying aesthetic experience in general.

## Figures and Tables

**Table 1:** Mean values of trait anxiety scores (TAI), perceived stress scores (PSS), and stress assessment in the last month, for the two samples.

Museum		N	Min	Max	Mean	SD
MAG	Trait Anxiety	17	25	61	44.57	9.37
	Perceived Stress	17	7	37	19.14	6.53
	Stress last month	17	3	10	7.14	1.91
NHM	Trait Anxiety	16	31	62	49.44	8.89
	Perceived Stress	16	10	32	20.78	5.84
	Stress last month	16	2	9	8.00	1.71

**Table 2:** *T*-tests for paired sample output for all of the dependent variables.

	Mean	SD	Df	*p*
SAI	−6.53	8.52	34	<0.001
Mood	1.17	1.11	34	<0.001
Stress	−1.67	1.96	34	<0.001
Mental Clarity	0.42	1.08	34	0.004
Contentment	1.08	1.26	34	<0.001
Calmness	1.00	1.50	34	<0.001
Restlessness	−71	2.60	34	0.030

**Table 3:** Descriptive statistics for the variables with significant pre/post effects for all of the dependent variables.

		Mean	SD
SAI	Pre	36.73	8.4
	Post	30.00	5.17
Mood	Pre	7.20	2.52
	Post	8.38	1.48
Stress	Pre	4.67	2.24
	Post	2.96	1.6
Calmness	Pre	7.24	1.24
	Post	8.28	0.96
Clarity of mind	Pre	7.76	1.58
	Post	8.20	1.16
Restlessness	Pre	3.37	1.88
	Post	2.62	1.28

**Table 4:** Linear regression model with ΔSAI as dependent variable.

	B	SE	Beta		*p*
(Constant)	−16.482	7.081		−2.328	0.033
ΔTBR	−807	0.242	−519	−3.333	0.004
PSS	−405	0.185	−354	−2.187	0.043
TAI	0.450	0.161	0.440	−2.802	0.012

## Data Availability

The data presented in this study are available upon request from the corresponding author. The data are not publicly available due to privacy concerns.
